# Hematological profile of pregnant women at St. Paul’s Hospital Millennium Medical College, Addis Ababa, Ethiopia

**DOI:** 10.1186/s12878-018-0111-6

**Published:** 2018-07-09

**Authors:** Angesom Gebreweld, Delayehu Bekele, Aster Tsegaye

**Affiliations:** 1 Department of Medical Laboratory Science, College of Medicine and Health Science, Wollo University, Dessie, Ethiopia; 2 Department of Obstetrics and Gynecology, Saint Paul’s Hospital Millennium Medical College, Addis Ababa, Ethiopia; 3 School of Medical Laboratory Science, College of Health science, Addis Ababa University, Addis Ababa, Ethiopia

**Keywords:** Pregnancy, Hematological profile, Anemia, Thrombocytopenia

## Abstract

**Background:**

In pregnancy, hematological changes occur in order to meet the demands of the developing fetus and placenta, with major alterations in blood volume. Abnormal hematological profile affects pregnancy and its outcome. This study aimed to assess hematological profiles of pregnant women at a tertiary care teaching hospital.

**Method:**

This cross sectional study was conducted among 284 consecutive pregnant women at St. Paul’s Hospital Millennium Medical College. Socio-demographic characteristics were collected using pre-tested structured questionnaire. About 4 ml of venous blood was collected from each participant for hematological parameters analysis using Cell-Dyn1800 (Abbott Laboratories Diagnostics Division, USA) and peripheral blood film review.

**Result:**

There were differences in mean hematological parameters between trimesters: specifically differences in mean values of WBC (1^st^and 3rd), Hb(1stand2^nd^ and 1^st^& 3rd), HCT (1^st^and2nd), RDW (1^st^and2^nd^ and 1^st^and3rd), neutrophil and lymphocyte (1stand 2nd and 1^st^and3^rd,^ for both) were statistically significant (*p* < 0.05). The prevalence rates of anemia and thrombocytopenia were 11.62 and 7.7%, respectively and were dominantly of mild type. On the bases of blood picture, we classified anemia’s of pregnancy as microcytic hypochromic (51.5%), normocytic hypochromic (27.3%), normocytic normochromic (18.2%), and dimorphic (3%).

**Conclusion:**

Significant changes in selected hematological parameters between trimesters, and an anemia and thrombocytopenia of mild type were documented in this study. The commonest morphologic features were mostly characteristic features of iron deficiency anemia. These warrant the need for monitoring hematological parameters of pregnant women at any stage of the pregnancy to avoid adverse outcomes.

**Electronic supplementary material:**

The online version of this article (10.1186/s12878-018-0111-6) contains supplementary material, which is available to authorized users.

## Background

In pregnancy, hematological changes occur in order to meet the demands of the developing fetus and placenta, with major alterations in blood volume. The plasma volume increase by 40 to 45% on average, this increase is mediated by a direct action of progesterone and estrogen on the kidney causing the release of renin and thus an activation of the aldosterone renin-angio-tensin mechanism. This leads to renal sodium retention and an increase in total body water. This increase occurs faster in the late second trimester [1–3].

Red blood cell mass increases by 15–20% as a result of the increase in the production of erythropoietin. As the increase in red cell mass is relatively smaller than that of plasma volume, the net result of hemoglobin (Hb) concentration falls by 1–2 g/dl. This is termed the physiological anemia of pregnancy [3, 4].

In pregnancy, the peripheral blood count of white blood cell (WBC) is raised due to pregnancy induced physiological stress. Neutrophils contribute most to the overall higher WBC count. [5]. However, the platelet count decreases during pregnancy because of hemodilution, increased platelet activation and consumption, particularly in the third trimester [4, 5].

Although physiological in nature, abnormal hematological profile affects pregnancy and its outcome. One of the most important underlying cause of maternal mortality is due to underlying hematological complications. Anemia and thrombocytopenia are the most frequent hematologic complications during pregnancy [6, 7].

Anemia of pregnancy is said to occur when Hb concentration is less than 110 g/l [8], as per World Health Organization (WHO) recommendation. Global prevalence of anemia in pregnant women is 41.8%. Africa and Asia are the most heavily affected regions. Throughout Africa, about 56% of pregnant women are anemic. As documented in the WHO2008 report, this hematological disorder is a severe public health problem in Ethiopian pregnant women and the estimated prevalence was 62.7% [9, 10].

The functional consequences of anemia are serious and include an increased risk of maternal, fetal, and neonatal mortality. Poor pregnancy outcomes such as low birth weight and preterm birth; impaired cognitive development, reduced learning capacity, and diminished school performance in children; and decreased productivity in adults are among the consequences [11]. In neighboring Sudan, 20.3% of maternal deaths are associated with anemia [12].

Thrombocytopenia is one of the most common hematologic abnormalities encountered during pregnancy. About 8–10% of pregnant women are affected by thrombocytopenia (platelet count < 150 × 10^9^/L), particularly in the third trimester. Approximately 75% of these cases are due to a benign process of gestational thrombocytopenia which is mild and have no significance for mother or fetus. But, in some instances, thrombocytopenia can also be associated with a complex clinical disorder such as preeclampsia and hemolysis, elevated liver enzymes, low platelets (HELLP) syndrome (20%), or idiopathic thrombocytopenic purpura (ITP) (5%). There can also be profound and even life-threatening results for both mother and baby [13–15].

As several studies showed pregnancy may have effect on hematological parameter and essential to monitor these parameters at any stage of the pregnancy [16–18]. This study was, therefore, conducted to assess hematological profile of pregnant women at St. Paul’s Hospital Millennium Medical College, Addis Ababa, Ethiopia. The study provided information about the magnitude of anemia, morphological type of anemia, thrombocytopenia and change of hematological values at different trimesters which is important to detect hematological complication early and to administer appropriate therapy.

## Methods

### Study design, area and setting

A cross sectional health facility based study was conducted at St. Paul’s Hospital, Addis Ababa, Ethiopia from June to August 2014. St. Paul’s Hospital Millennium Medical College (SPHMMC) is the second largest public hospital in Ethiopia, which is located in Gullele sub city in Addis Ababa and built by Emperor Haile Selassie in 1969. The hospital receives referrals from around the country and is under the guidance of the Ethiopian Federal Ministry of Health.

### Population

A total of 284 consecutive pregnant women were enrolled from antenatal care clinic of obstetrics and gynecology Department of SPHMMC. Written informed consent was obtained from all. Pregnant women with bleeding problem, multiple pregnancies, Hepatitis B Virus infection, human immunodeficiency virus and less than 18 years of age were excluded from the study.

### Data collection

A structured pre tested interviewer administered questionnaire (see Additional file 1) and medical records were used to collect socio-demographic and clinical data of the study participants. Venous blood specimen (4 ml) was taken from each pregnant woman by a senior laboratory professional for peripheral blood film and complete blood count. Cell-Dyn 1800 (Abbott Laboratories Diagnostics Division, USA) hematological analyzer was used to determine complete blood count. Peripheral blood smear were prepared and stained by Wright’s stain to look at morphological characteristics of anemia. The peripheral smears were examined by a senior laboratory technologist and principal investigator independently. Standard operating procedures were strictly followed in each step to maintain quality of the laboratory results.

According to WHO, Anemia of pregnancy is said to occur when Hb concentration is less than 110 g/l. Anemic pregnant women were further categorized as women with mild anemia, moderate anemia and severe anemia which corresponds to Hb value 100–109 g/l, 70–99 g/l, and lower than 70 g/l respectively [8]. Thrombocytopenia is said to be present when the platelet count of the pregnant women is less than 150 × 10^9^ / L. The platelet counts from 100 to 150 × 10^9^/L is considered mild thrombocytopenia, levels ranging from 50 to 100 × 10^9^/L are considered as moderate thrombocytopenia and levels less than 50 × 10^9^/L are considered as severe thrombocytopenia [15].

### Data analysis

The data was entered and analyzed using Statistical Package for the Social Science (SPSS) Version16 statistical software. Frequencies and means ± standard deviation (SD) were used to summarize descriptive statistics. One-way analysis of variance (ANOVA) was used in the analysis to compare the hematologic values among trimesters. *P* values < 0.05 were considered as statistically significant.

### Ethical considerations

The study was approved by Departmental Research and Ethics Review Committee (DRERC) of the Department of Medical Laboratory Sciences, Addis Ababa University. After a letter of cooperation sent to St Paul’s Hospital Millennium Medical College from the Department of Medical Laboratory Sciences the Institutional Review Board also approved the study. Then a letter informing the hospital administrators was written from the Institutional Review Board (IRB) and Permission obtained from St. Paul’s Hospital Millennium Medical College to conduct the study. Individual consent was obtained before the questionnaires were administered and blood samples were collected. To ensure confidentiality, participants’ data were linked to a code number. Any abnormal test results of participants were communicated to their attending physician.

## Results

### General characteristics of the study participants

A total of 284 pregnant women with a mean (SD) age of 27.3 ± 4.48 years (ranges from 18 to 40) were included in the study. About 170 (59.9%) were in their third trimester, 66 (23.2%) in second trimester, and 48 (16.9%) in first trimester. Majority of the study groups 118 (41.5%) were in the age range of 26–30 years and urban residents (261, 91.9%) (Table 1).

**Table 1:** Characteristics of Pregnant women (*N* = 284) at St. Paul’s Hospital Millennium Medical College Addis Ababa, Ethiopia, June to August 2014

Variables		Frequency	Percentage (%)
Age group (years)	≤20	16	5.6
	21–25	93	32.7
	26–30	118	41.5
	31–35	43	15.1
	≥36	14	4.9
Occupation	Farmer	16	5.6
	Housewife	164	57.7
	Government	24	8.5
	Student	8	2.8
	Private	72	25.4
Educational status	Illiterate	42	14.8
	Elementary	115	40.5
	Secondary	54	19.0
	Preparatory	23	8.1
	University/college	50	17.6
Residence	Rural	23	8.1
	Urban	261	91.9
Trimester	1st trimester	48	16.9
	2nd trimester	66	23.2
	3rd trimester	170	59.9

### Hematological profiles of the study participants

The overall mean (SD) of selected hematological parameters for the study participants were as follows: WBC count 7.93 ± 2.68 × 10^9^/L, RBC count 4.58 ± 2.34 ×  10^12^/L, Hb130.1 ± 16.4 g/L, HCT 40.07 ± 4.15%, MCV 90.60 ± 6.59 fL, MCH 29.32 ± 2.72 pg, MCHC 32.33 ± 1.35%, and PLT 249.36 ± 80.08 × 10^9^/L(Table 2).

**Table 2:** Hematological Profiles of pregnant women based on trimesters (Mean ± SD) in St. Paul’s Hospital Millennium Medical College Addis Ababa, Ethiopia, June to August 2014

Parameters	Trimester				*P*-Value		
	Overall	1^st^ trimester PW	2^nd^ trimester PW	3^rd^ trimester PW	1^st^&2^nd^	1^st^&3^rd^	2^nd^&3^3rd^
WBC x 10^9^/L	7.93 ± 2.68	7.02 ± 2.61	7.83 ± 2.62	8.22 ± 2.68	.246	.018	.580
RBC × 10^12^/L	4.58 ± 2.34	4.61 ± 0.51	4.86 ± 4.78	4.46 ± 0.47	.836	.927	.475
Hb (g/l)	130.1 ± 16.4	136.5 ± 15.9	126.2 ± 17.2	129.7 ± 15.8	.002	.031	.275
HCT (%)	40.07 ± 4.15	41.59 ± 4.47	38.92 ± 4.47	40.08 ± 3.79	.002	.061	.126
MCV (fl)	90.60 ± 6.59	90.26 ± 5.68	91.24 ± 7.29	90.45 ± 6.56	.712	.982	.689
MCH (pg)	29.32 ± 2.72	29.58 ± 2.41	29.53 ± 3.16	29.16 ± 2.62	.995	.615	.618
MCHC %	32.33 ± 1.35	32.72 ± 1.42	32.30 ± 1.51	32.23 ± 1.24	.229	.070	.934
RDW (%)	13.99 ± 1.71	13.25 ± 1.22	14.51 ± 2.09	14.01 ± 1.59	.000	.017	.099
PLT x 10^9^/L	249.36 ± 80.08	267.62 ± 100.89	254.02 ± 68.06	242.39 ± 77.29	.641	.131	.575
MPV (fl)	9.62 ± 1.37	9.48 ± 1.32	9.60 ± 1.61	9.66 ± 1.28	.888	.708	.957
Lymphocyte (%)	24.31 ± 8.64	28.42 ± 10.68	24.22 ± 8.16	23.18 ± 7.84	.025	.001	.674
MID WBC (%)	7.57 ± 2.34	8.07 ± 2.5	7.11 ± 2.12	7.61 ± 2.35	.986	.863	.707
Neutrophil (%)	67.72 ± 9.17	63.52 ± 11.27	67.91 ± 8.33	68.82 ± 8.52	.028	.001	.766

*PW* pregnant women, *P* < 0.05 is statically significant

MID WBC: which include Monocyte, eosinophile, basophile, and other midsized immature WBCs

When analyzed by trimester, the mean (SD) WBC values for the respective first, second and third trimester pregnant women were 7.02 ± 2.61, 7.83 ± 2.62, and 8.22 ± 2.68(× 10^9^/L), respectively. The difference was statistically significant between those in1^st^ and 3^rd^ trimester (*P* < 0.05).The Mean Hb value of pregnant women in first trimester (136.5 ± 15.9 g/l) was significantly higher compared to those in second trimester (126.2 ± 17.2 g/l), and in third trimester (129.7 ± 15.8 g/l). Mean HCT value in the three pregnancy groups were 41.59 ± 4.47, 38.92 ± 4.47, and 40.08 ± 3.79 (%), with a statistically significant difference between those in 1^st^ and 2^nd^ trimesters (*P* < 0.05). Whereas the mean red cell indices (MCV, MCH and MCHC) and mean PLT values did not differ between the three trimester groups (Table 2).

Moreover, the mean RDW values of those in the 2^nd^ and 3^rd^ trimesters are higher than those in the 1^st^ trimester. The neutrophil counts also follow the same increasing pattern while the lymphocyte counts in the 2^nd^ and 3^rd^ trimesters group are significantly lower than those in the 1st trimester (*P* < 0.05) (Table 2).

### Hematological abnormalities

**Fig. 1:**
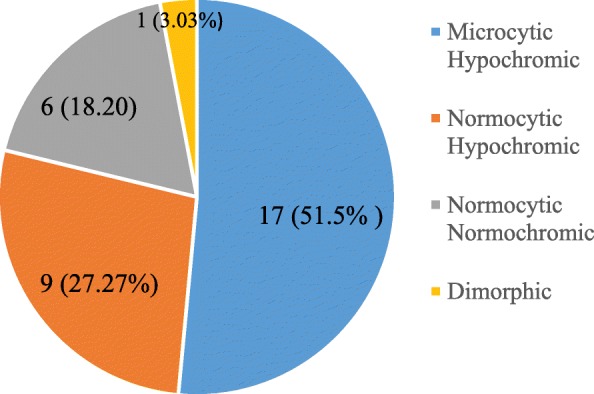
Distribution of Morphologic Type of Anemia among the anemic pregnant women (*n* = 33), St. Paul’s Hospital Millennium Medical College Addis Ababa, Ethiopia, 2014

Using the WHO criterion of Hb < 110 g/dl as indicative of anemia, 33 (11.62%) pregnant mothers were anemic. Of them, 23 (69.70%) were mildly anemic (Table 3). Based on RBC morphologic classification of anemia, most of the anemic pregnant women had microcytic hypochromic 17 (51.5%) type of anemia (Fig. 1).

**Table 3:** Distribution of anemia by severity among the anemic pregnant women (*n* = 33), St. Paul’s Hospital Millennium Medical College Addis Ababa, Ethiopia, 2014

Severity of anemia	Number	Percentage (%)
Mild anemia	23	69.7
Moderate anemia	10	30.3
Severe anemia	0	0
Total	33	100

Thrombocytopenia (Platelet count < 150 × 10^9^/L), was detected in 22 pregnant women giving a prevalence of 7.7%. Among them, most 20 (90.91%) were mildly thrombocytopenic (Table 4). The prevalence of thrombocytopenia was 4.2, 6.1 and 9.4% at first, second and third trimester groups, respectively (Table 5).

**Table 4:** Distribution of thrombocytopenia by severity among thrombocytopenic pregnant women (*n* = 22), St. Paul’s Hospital Millennium Medical College Addis Ababa, Ethiopia, 2014

Severity	Number	Percentage (%)
Mild thrombocytopenia	20	90.91
Moderate thrombocytopenia	2	9.09
Severe thrombocytopenia	0	0
Total	22	100

**Table 5:** Distribution of thrombocytopenia among pregnant women at different trimesters (*N* = 284), St. Paul’s Hospital Millennium Medical College Addis Ababa, Ethiopia, 2014

Characteristics	Thrombocytopenia status		Total
	Thrombocytopenic (%)	Non-Thrombocytopenic (%)	
Trimester			
1st trimester	2 (4.2%)	46 (95.8%)	48
2nd trimester	4 (6.1%)	62 (93.9%)	66
3rd trimester	16 (9.4%)	154 (90.6%)	170

## Discussion

The study reported herein aimed to determine the hematological profile of pregnant women visiting St. Paul’s Hospital Millennium Medical College in Addis Ababa from June to August 2014.

The progressive increment of WBC from those in their first (7.02 ± 2.61) to those in their third (8.22 ± 2.68) trimester and the dominance of neutrophil in our study is consistent with findings of Akinbami et al. (from 7.37 ± 2.38 to 8.31 ± 2.15) [19], Das et al. (from 6.14 ± 1.76 to 8.09 ± 4.12) [16], Osonuga et al. (from 6.22 ± 1.79 to 8.11 ± 4.13) [17] and Ifeanyi et al. (from4.8+/− 2.6 to 7.81+/− 1.7) [18]. Physiologic stress induced by pregnancy [5] has been implicated as a possible mechanism for pregnancy associated leukocytosis. Besides, fetal immunity development pathways which include selective immune tolerance and modulation have also been suggested as possible explanations [20].

The finding of a significantly higher number of neutrophils in the second and third trimester pregnant women compared to the first trimester pregnant women in our study concurs with this scientific explanation. Neutrophils are the major type of WBC counts and their number can double during pregnancy compared to its postpartum values [5, 6].

In the present study, hemoglobin concentration and hematocrit values were highest in the first trimester, reach their lowest point in the second trimester and begin to raise again in the third trimester groups. This is consistent with a study conducted by James et al. (Hb127.3 ± 11.4114.1 ± 11.6, & 116.7 ± 11.8 g/l and HCT 37.05 ± 2.96, 33.12 ± 3.00 and 34.03 ± 2.97% for 1st, 2nd and 3rd trimesters respectively) [21] and Akinbami et al. (32.07 ± 6.80, 29.76 ± 5.21, and 33.04 ± 3.88%) [19] for hematocrit. While it contradicts with a study conducted by Ifeanyi et al. [18] and Osonuga et al. [17] in Nigeria which respectively showed low Hb and HCT in the first trimester, highest in the second trimester and drop in the 3rd trimester.

The decrease in hemoglobin concentration and packed cell volume from those in first trimester to those in second trimester may be due to hemodilution, hormonal changes, and increased iron demand [6, 16, 19]. Hormonal changes results production of rennin from kidneys to increase plasma volume during pregnancy. The increase in plasma volume is relatively greater than the increase in red cell mass, which results in a fall in maternal Hb and HCT. In late pregnancy, plasma volume increases slowly that lead to a slight rise in hemoglobin and hematocrit value, it may account for the slight rise in Hb and HCT in the third trimester [5, 19].

Our study also reported a gradual reduction in PLT count as pregnancy advanced but the mean difference between the three trimesters was not statically significant. Our finding is similar with study conducted by Ajibola et al. [22], Akinbami et al. [19] and James et al. [21]. The reduction of platelet count as pregnancy advanced may be due to an increase in blood volume, increased platelet activation, and decreased life span in the uteroplacental circulation [5–7]. The present study also found an increment of mean platelet volume as the pregnancy advanced. This result is in agreement with a study conducted in Port Harcourt, Nigeria [23].

The finding of 11.62% anemia in this study is comparable to studies conducted in Iranian pregnant women (13.6%), Nakhonsawan, Thailand (14.1%), Sudaneese (10%), and Ethiopian women from Hawassa (15.1%), Gondar (16.6%), and Debre Berhan (9.7%) [24–29].

The result of the present study is much lower than studies conducted in Karantaka India (82.9%), highlands of Tibet (ranges 41.3–77.9%), Nepal (41.02%), Uyo Nigeria (54.5%), Jamaica (34.8%), west Algeria (40.08%), Uganda (63.1%), Eastern Ethiopia (56.8%), south west Ethiopia (53.9%), and Arsi zone (Ethiopia) (36.6%) [30–39]. Our result is also lower than results reported by studies in Turkey (27.1%), Sokoto, Nigeria (21.3%), and two other studies from Ethiopia namely Azezo in Gondar (21.6%), and Tikur Anbessa Specialized Hospital in Addis Ababa (21.3%) [40–43].

The possible reason for the difference may be due to the differences in socio economic status, geographical variation and differences in dietary habits of the study participants. The lower result of our study may also be due to the Governments effort to achieve Sustainable Development Goals (SDGs).

The predominance of mild type of anemia in the current study fit well with studies conducted in Uyo Teaching Hospital Nigeria [33], Western Nepal [32], and studies conducted in different parts of Ethiopia: Tikur Anbessa Specialized Hospital [43], Debre Berhan Health Institutions [29], Southwest Ethiopia [38], and Gondar [28]. However, our result deviates from the findings from Karnataka India, west Algeria and Jimma (Ethiopia) which showed high rate of moderate Anemia [30, 35, 44].

The common morphological characteristic of anemia identified in our study, mainly microcytic hypochromic, and normocytic hypochromic, is deviated from studies conducted in Turkey [40], Northern Nigeria [45] and Gondar [28] which showed higher rate of normocytic normochromic type of anemia. Microcytic hypochromic and normocytic hypochromic blood picture are characteristic of iron deficiency anemia [33, 46], and our findings are in agreement with studies conducted in Sudan [47], Sokoto Nigeria [41], Uyo Nigeria [33], and New Delhi [46].

Thrombocytopenia is second to anemia as the most common hematologic abnormality encountered during pregnancy. The finding of 7.7% thrombocytopenia prevalence in the current study is similar to studies conducted in India (8.17%) and (8.8%), Iraq (8%), and Ahmedabad (7.67%) [48–51]. It also agrees with values indicated in a literature review conducted by Myers [15], which showed 8–10% rate of thrombocytopenia of all pregnancies. However, our result is lower than studies conducted in Ghana (15.3%) and Nigeria (13.5%) [22, 52].

The mildness of thrombocytopenia noted in the current study parallels findings from Iraq [51] Ghana, India, Nigeria, and Ahmedabad [22, 48, 49, 52]. The finding of predominantly mild thrombocytopeniamay be attributed to gestational thrombocytopenia (GT), which is of mild type and accounts for the majority of thrombocytopenias during pregnancy [7]. Though it is not associated with any adverse events for either the mother or baby and requires no specific treatment, other etiologies must be excluded (i.e. megaloblastic anemia, immune thrombocytopenia, eclampsia, and liver disorders) [6]. Especially, many features of GT are similar to mild immune thrombocytopenia and it can be difficult to distinguish between the two disorders [15].

The observed high prevalence of thrombocytopenia in the third trimester, which also agrees with other studies [22, 49, 51, 52], could be due to an increase in platelet aggregation especially during last 8 weeks of gestation. It has been reported that significant fall in platelet count can occur from 32 weeks of gestation onwards [6]. In the third trimester, platelet count decreases due to hemodilution, increased platelet activation and consumption [5].

## Conclusion

In conclusion, in this study WBC, Hb, HCT, RDW, lymphocyte and neutrophil counts showed statistically significant difference between trimesters (*P* < 0.05). The prevalence of anemia and thrombocytopenia, both predominantly of mild type, were 11.62 and 7.7%, respectively. Therefore, the pregnant women should be monitored and their hematological parameters properly interpreted to recognize and avoid pregnancy complications early. This will be of paramount importance in line with meeting the SDGs target related to maternal and child health.

## Additional file

Additional file 1: Questionnaires. The data within additional file 1 contains questionnaires, which were used to collect information from the study participants for this study. The questionnaires had two parts; the first part is for collecting data about socio-demographic characteristics of the study subjects. The second part is for collecting complete blood count of the study participants. (DOCX 26 kb)

## Abbreviations

ANC: Antenatal care
EDTA: Ethylene DiamineTetra Acetic acid
GT: Gestational thrombocytopenia
Hb: Hemoglobin
HCT: Hematocrit
MCH: Mean cell hemoglobin
MCHC: Mean cell hemoglobin concentration
MCV: Mean cell volume
MPV: Mean platelet volume
PLT: Platelet
RBC: Red blood cell
RDW: Red cell distribution width
SDGs: Sustainable Development Goals
WBC: White blood cell
WHO: World health organization
