# Supplementary material for: Hematological profile of pregnant women at St. Paul’s Hospital Millennium Medical College, Addis Ababa, Ethiopia
Source: BMC Hematol. 2018 Jul 9;18:15. doi: 10.1186/s12878-018-0111-6 (PMC6038189; doi:10.1186/s12878-018-0111-6)
Supplement: Supplementary file 1 — Questionnaires. The data within additional file 1 contains questionnaires, which were used to collect information from the study participants for this study. The questionnaires had two parts; the first part is for collecting data about socio-demographic characteristics of the study subjects. The second part is for collecting complete blood count of the study participants. (DOCX 26 kb) [file 12878_2018_111_MOESM1_ESM.docx]

**Hematological profile of pregnant women at St. Paul’s Hospital Millennium Medical College, Addis Ababa, Ethiopia.**

***English Version Questionnaire***

**Part I: Questionnaire about Socio demographic characteristics of Pregnant Women**

Serial number__________ ID. No.___________________ Date___________

1. How old are you? ____________________
2. Where do you live?(Residence)
3. Urban
4. Rural
5. What is your occupation?
6. Farmer
7. Housewife
8. Student
9. Private employee
10. Governmental employee
11. What is your educational Status?
12. Illiterate
13. Primary School
14. Secondary school
15. Preparatory school
16. University/college graduate
17. What is your Gestational age in weeks? ____________________

**Part II: Laboratory result of Pregnant Women**

1. Complete blood count result (attach the print out with this questioner or fill the result on the provided space below carefully)

| A. | WBCx10^9^/L___________ | H. | Neutrophile(%)_____________ |
| --- | --- | --- | --- |
| B. | RBCx10^12^/L___________ | I. | Lymphocyte (%) ___________ |
| C. | Hb(g/l)_______________ | J | MID WBC(%)______________ |
| D. | HCT (%)______________ | K | RDW(%)______________ |
| E. | MCV(fl)______________ | L | PLTx10^9^/L_____________ |
| F. | MCH(pg)_____________ | M | MPV(fl)_______________ |
| G. | MCHC (%)____________ |  |  |

Signature of the data collector: _____________ Date____/____/_____

Checked by supervisor:

Signature________________ Date ____/___/______
